# miR160: An Indispensable Regulator in Plant

**DOI:** 10.3389/fpls.2022.833322

**Published:** 2022-03-22

**Authors:** Kai Hao, Yun Wang, Zhanpin Zhu, Yu Wu, Ruibing Chen, Lei Zhang

**Affiliations:** ^1^Department of Pharmaceutical Botany, School of Pharmacy, Naval Medical University, Shanghai, China; ^2^Biomedical Innovation R&D Center, School of Medicine, Shanghai University, Shanghai, China; ^3^Institute of Interdisciplinary Integrative Medicine Research, Medical School of Nantong University, Nantong, China; ^4^Shanghai Key Laboratory for Pharmaceutical Metabolite Research, Shanghai, China

**Keywords:** miR160, *ARFs*, growth and development, stress response, secondary metabolism

## Abstract

MicroRNAs (miRNA), recognized as crucial regulators of gene expression at the posttranscriptional level, have been found to be involved in the biological processes of plants. Some miRNAs are up- or down-regulated during plant development, stress response, and secondary metabolism. Over the past few years, it has been proved that miR160 is directly related to the developments of different tissues and organs in multifarious species, as well as plant–environment interactions. This review highlights the recent progress on the contributions of the miR160-ARF module to important traits of plants and the role of miR160-centered gene regulatory network in coordinating growth with endogenous and environmental factors. The manipulation of miR160-guided gene regulation may provide a new method to engineer plants with improved adaptability and yield.

## Introduction

MicroRNAs (miRNA) are a class of 20–24 nt small non-coding single-stranded RNAs which are found with high conservation and are widely presented in eukaryotes (Ambros et al., 2003; Bartel, 2004). In plants, RNA polymerase II participates in miRNAs transcription to derive pri-miRNAs, which can fold to form a hairpin structure and be processed by Dicer-like RNase III endonucleases (DCLs). The pri-miRNAs are cut by DCL1/HYL1/SE to produce most of the miRNAs in the nucleus through four processing pathways (short base to loop, sequential base to loop, short loop to base, and sequential loop to base), other DCLs can also be involved in miRNA production (Bologna et al., 2013; Moro et al., 2018; Li and Ren, 2021). The nascent miRNA:miRNA* duplexes generated by DCL-mediated processing will be methylated at the 3′-ends by HUAENHANCER 1 (HEN1). Recent study shows that mature miRNA is mainly bound to the ARGONAUTE (AGO) in RNA-induced silencing complex (RISC) in the nucleus and exported to the cytosol by EXPO1. But some miRNA:miRNA* duplexes may be exported by HASTY and assembled in the cytosol (Jones-Rhoades et al., 2006; Bologna et al., 2018; Song et al., 2019; Wang et al., 2019; Brioudes et al., 2021; Cambiagno et al., 2021). Mature miRNAs recognize target messenger RNA (mRNA) sites by perfect or near perfect complementarity, then it can regulate mRNA expression negatively by cleaving it or repressing translation. The correct temporal and spatial accumulation of some highly conserved miRNAs is essential for maintaining the normal development of plants. miR160 is one of miRNAs that regulate the auxin signaling pathways and plays a critical role in various biological processes of plants. Here we reviewed the structures of miR160 family, their targets, expression patterns, and functions in plant development, abiotic/biotic responses, and secondary metabolism.

## The miR160 Family and *AUXIN RESPONSE FACTORS*

MiR160 is a conserved miRNA that is popularly confirmed in many model and non-model plants, such as *Arabidopsis*, tomato, rice, poplar, and so on. The first miR160 was identified in *Arabidopsis* and the miR160 family was encoded by three loci (Reinhart et al., 2002). *MIR160b* and *MIR160c* were very similar, but they were different from *MIR160a* (Liu et al., 2010). Recently, a large number of *MIR160* homologs have been discovered in land plants and *Brassicaceae*. *MIR160a* was considered to be the progenitor of *MIR160b* and *MIR160c* paralogs because they originated from the segmental duplication of the region encompassing the ancient prototype *MIR160a* (Singh and Singh, 2021). The miR160 family has tissue-specific expression due to various functions. In tobacco, miR160 was expressed flower buds and vascular bundles, but no expression signal could be detected in young leaves and seeds (Valoczi et al., 2006). The miR160 family in plants targets the *AUXIN RESPONSE FACTORS* (*ARFs*) transcription factors which have been found in the auxin signaling pathways (Li et al., 2016). *ARF* specifically binds to auxin-responsive elements (AuxREs) *TGTCTC* in the promoter region of auxin-responsive genes to activate or inhibit gene expression (Tiwari et al., 2003). It can also combine with Aux/indoleacetic acid (IAA) inhibitors to form dimers which are regulated by auxin.

In *Arabidopsis*, the *ARF* gene families consisting of 23 loci had been identified to date (Remington et al., 2004). It was reported that miR160 could regulate a group of *ARF* genes: *ARF10*, *ARF16*, and *ARF17* (Rhoades et al., 2002). They all had a fragment that could be recognized by miR160 in a conserved *N*-terminal DNA binding domain (DBD). But *ARF17* was different from others because of its poorly conserved *C*-terminal dimerization domain (CTD) (Tiwari et al., 2003). Recent studies demonstrated that three genes had a significant impact on the development of *Arabidopsis*. For instance, the increased levels of *ARF17* due to the missing of miR160 regulation could contribute to severe developmental abnormalities, such as defects in vegetative, adventitious root (AR), embryonic, and floral development (Sorin et al., 2005). In addition, *OsARF18* was found as the target of miR160 in rice (Huang et al., 2016). Many results show that miR160 and its target gene *ARF* family are important to plant development, anabolism, and abiotic stress.

## Regulate the Development of Plants

**FIGURE 1:**
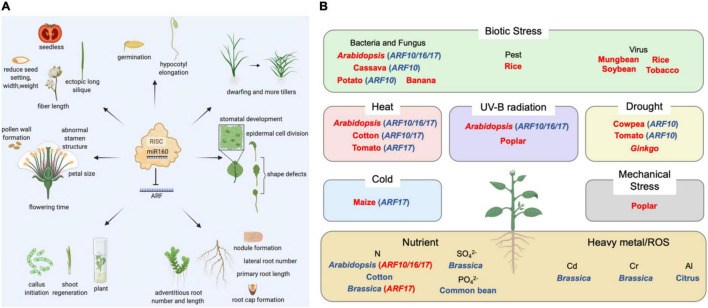
The functions of miR160-ARF module in plants. **(A)** List of miR160-ARF’s role in various plants during their growth and development. **(B)** Summary of plant stress responses with miR160 involvement. Blue represents gene down-regulation; red represents gene up-regulation.

The miR160 family targets the *ARF* gene involved in the auxin signal transduction pathway, and it is essential for the growth and development of plants (Figure 1A). Different *ARF* genes mediate auxin signals in different parts, thereby controlling the fate of cell differentiation. *ARF* is also of great significance for mediating the interaction between auxin and other hormones.

### Seed Germination

In *Arabidopsis*, transgenic seeds overexpressing miR160 were less sensitive to abscisic acid (ABA) during germination, but miR160-resistant (*mARF10*) mutant seeds were hypersensitive to ABA and impaired seedling establishment. The negative regulation of *ARF10* by miR160 was crucial to seed germination and post-embryonic development through involving the interactions between *ARF10*-dependent auxin and ABA pathways (Liu et al., 2007). Moreover, auxin controlled the expression of ABI3 and dramatically released seed dormancy by recruiting the *ARF10* and *ARF16* during seed germination (Liu et al., 2013). By analyzing the phenotypes of the MIR160a loss-of-function mutants without a 3′ regulatory region, MIR160a and its targets *ARF10/16/17* fulfilled a key role in embryo development. Auxin took part in regulating the expression of MIR160a by its 3′ region (Liu et al., 2010). miR160-*ARF10/16/17* were also found as a modulator in the cross-talk of auxin, light, gibberellin (GA), and brassinosteroids (BR) during hypocotyl elongation of *Arabidopsis*. Among them, *ARF10* was associated with GA (Dai et al., 2021).

### Root Development

The miR160-*ARF* module has been shown to affect the root development of plants (Barrera-Rojas et al., 2021). In *Arabidopsis*, *ARF17* as a regulator of auxin-inducible *GH3-like* mRNAs altered the primary root length and lateral root (LR) number (Mallory et al., 2005). *ARF10* and *ARF16* were considered as a whole controlled root cap formation, although their functions were redundant. In addition, the regulation of *ARF16* expression by miR160 was essential for maintaining the LR production (Wang et al., 2005). The same phenomenon was observed in methane-induced tomato LR formation (Zhao et al., 2019). In apple rootstock, over-expressed miR160a reduced *ARF16/17* levels and inhibited AR formation, including the number and length (Meng et al., 2020). The miR160-*ARF17* module was also an important regulator in the development of AR of poplar and lotus (Libao et al., 2020; Liu et al., 2020). It is important to control the auxin/cytokinin balance during nodule development. In soybean, miR160 negatively regulated *ARF10/16/17*, and overexpression of miR160 led to hyposensitivity to cytokinin and auxin hypersensitivity, and reduced nodule primordium initiation (Turner et al., 2013). At the later stage of nodule development, high miR160 activity favored auxin activity and promoted the nodule maturation albeit in spite of reduced nodule formation (Nizampatnam et al., 2015). The feedback regulatory loops involving miR160/*ARF* and auxin/cytokinin governed root and nodule organogenesis, which were also presented in *Medicago* (Bustos-Sanmamed et al., 2013).

### Shoot and Shoot Lateral Organ Growth

Evidence shows that miR160 plays an important role in the normal growth of leaves. In *Arabidopsis*, the overexpression of *mARF* (miR160-resistant) or *eTM160* (inactivation of miR160) increased the accumulation of *ARFs* and caused severe leaf developmental defects, including leaf shape defects (serrated leaves) and leaf symmetry anomalies (Mallory et al., 2005; Wu et al., 2013). In tomatoes, miR160 was required for leaflet initiation and controlled the final leaf shape and structure. Both the miR160-targeted *ARFs* and the Aux/IAA protein SlIAA9/ENTIRE were required to locally inhibit lamellipodia growth between initiating leaflets. They reduced lamina, increased leaf complexity, and decreased auxin response in young leaves (Hendelman et al., 2012; Ben-Gera et al., 2016; Damodharan et al., 2016). Among them, *ARF10A* was proved indispensable by the knockout of miR160-targeted *ARFs*, while the functions of *ARF16A* and *ARF17* were redundant (Damodharan et al., 2018). *SlARF10* also influenced stomatal development and ABA synthesis/signal response so as to close the stomata under drought. In addition, *SlARF10* enhanced hydraulic conductance by directly increasing aquaporin expression. An appropriate *ARF10* expression level controlled by miR160 was important to keep the leaf water balance between leaf development and adaptation to water stress (Liu X. et al., 2016). Moreover, miR160 controlled leaf curvature in potatoes by modulating the activity of *StTCP4* (involved in cell differentiation) and *StCYCLIND3;2* (involved in cell proliferation). The leaves of plants with overexpressed miR160 had a high positive curvature because of prolonged activation of the cell cycle in the center region of leaves. However, the leaves of miR160-knockdown plants were flattened, the *StTCP4* activity in both the central and marginal regions of leaves could be responsible for the flattened leaf phenotype (Natarajan and Banerjee, 2020). But the over-accumulation of *ARF18* showed rolled leaves in rice due to the anomaly in epidermal cell division in leaves (Huang et al., 2016).

Furthermore, miR160 decreased the accumulation of *ARFs* in the stem, reduced plant dwarfing, and formed more tillers by fine-tuning auxin signaling in rice (Huang et al., 2016).

### Reproductive Development

MiR160 controlled the floral organ growth by targeting *ARFs*, especially *ARF17*, in *Arabidopsis*. The mutants expressing *mARF17* had dramatic floral developmental defects, including accelerated flowering time, reduced petal size, altered phyllotaxy along the primary and lateral stems, abnormal stamen structure, and sterility (Mallory et al., 2005). Auxin was vital in plant male reproductive development (Cecchetti et al., 2008; Cardarelli and Cecchetti, 2014). The biosynthesis and transport of auxin were related to anther and pollen formation (Bender et al., 2013). The response of *ARF17* to auxin affected both abnormal stamen structure and male sterility, the overexpression of *ARF17* led to defects in microsporocytes and tapetum (Wang et al., 2017). But rational *ARF17* was essential to pollen wall formation pollen tube growth (Yang et al., 2013). The miR160-*ARF17* module associated with anther and pollen development had been found in many other species, such as cotton, tobacco, tomato, and maize (Valoczi et al., 2006; Li et al., 2019; Chen et al., 2020; Keller et al., 2020). In tomatoes, miR160 regulated auxin-mediated floral organ patterning and abscission (Damodharan et al., 2016, 2018).

The miR160-*ARF* module has a certain regulatory influence on the fruits and seed development of plants. In *Arabidopsis*, the *mARF10* plants produced ectopic siliques with wavy silique walls (Liu et al., 2007). Furthermore, the overexpression of miR160 repressed the *ARF10/17* and increased the silique length in *Brassica napus* (Chen et al., 2018). In tomatoes, overexpressing *mARF10* changed the early development of fruits and seeds, resulting in cone-shaped and/or seedless fruit (Hendelman et al., 2012). miR160 negatively regulated *ARF17* and the related gene *GH3*, resulting in the increase of indole-3-acetic acid that is involved in fiber elongation. Finally, the fiber length of cotton was increased (Liu et al., 2019). In rice, *ARF18* decreased seed setting, seed width, and seed weight. At the same time, the starch accumulation was significantly reduced (Huang et al., 2016). miR160 inhibited *ARF10/16/17*, also contributed to the assimilation and allocation of sulfate during seed filling in *Phaseolus vulgaris* L. (Parreira et al., 2021).

While specific mechanisms are still unclear for many plants, detailed developmental research of various *mARFs* mutants in *Arabidopsis* has revealed the contribution of specific family members to reproductive development, proving that they are indispensable for stamen development.

### Regeneration Process

Auxin and cytokinin are significantly involved in the regeneration process. In *Arabidopsis*, miR160 repressed callus initiation and shoot regeneration by cutting *ARF10* which was bounded to the *ARR15* promoter, repressed *ARR15* expression, and promoted the cytokinin response (Qiao et al., 2012; Qiao and Xiang, 2013; Liu Z. et al., 2016). Being essential in LEC2-mediated somatic embryogenesis (SE), the auxin signaling pathway was controlled by miR160 and *ARF10/16/17* of *Arabidopsis*. The repression of miR160 led to the spontaneous formation of somatic embryos and the significant accumulation of auxin in the cultured explants (Wojcik et al., 2017). The similar effects of miR160-*ARF16/17* auxin signal transduction could be observed by target mimicry technology during the early stages of longan SE (Lin et al., 2015). At the late embryogenic calli callus induction stages of maize, *ARF19* was significantly increased with miR160 being heavily reduced, but their roles in regeneration remained unclear (Lopez-Ruiz et al., 2019).

## Role of the miR160 in the Interaction of Plants With the Environment

### Abiotic Stress

Abiotic stress is caused by the change of external natural conditions, including cold, heat, drought, high light, mechanical stress, salinity, metals (including heavy metals), nutrient deficiencies, and so on. In the wild, plants are often subjected to a combination of abiotic stresses (Mittler, 2006). It has been found that miR160 is directly associated with plant responses to several stress conditions.

In *Arabidopsis*, miR160 suppressed *ARF10/16/17* expressions to control heat shock protein (HSP) genes levels and regulate thermotolerance (Li et al., 2014; Lin et al., 2018). Accumulating evidence supports a role of the miR160-*ARF* network in male sterility caused by long-term high temperature (HT) stress. Overexpressing miR160 increased cotton sensitivity to HT stress with the decrease of *ARF10/17* mRNA levels, resulting in another indehiscence by activating the auxin response at the sporogenous cell proliferation stage (Ding et al., 2017; Chen et al., 2020). miR160 and *ARF17* also regulated the development of transition from post-meiotic to mature pollen in tomatoes under heat stress response (Keller et al., 2020). This gene pair were found to participate in the response to chilling stress in maize as well (Aydinoglu, 2020).

The expressions of several conserved miRNAs were analyzed in two cowpea genotypes during water deficit. miR160a/b has been found strongly upregulated, which inversely correlated to the expression levels of their targets *ARF10* (Barrera-Figueroa et al., 2011). In *Ginkgo biloba*, miR160a decreased *ARFs* expression and took part in the drought stress tolerance through the IAA signaling pathway (Chang et al., 2020). It is possible that the increase of miR160 and the concomitant decrease of *ARF* are part of the plant’s strategy to balance water loss and growth under drought.

The exposure to ultraviolet-B (UV-B) can generally cause irreversible damage to DNA, proteins and lipids, and overwhelms antioxidant defense systems in plants because of the excessive production of reactive oxygen species (ROS). A total of 11 putative UV-B-responsive miRNAs, including miR160, were identified by UV-B radiation in *Arabidopsis* (Zhou et al., 2007). In addition, a set of miRNAs that were responsive to UV-B radiation were found by miRNA filter array assay in *P. tremula*. miR160 was one of 13 up-regulated miRNAs by UV-B radiation (Jia et al., 2009).

In poplar, miR160 belonged to a pair of mechanical stress-induced miRNAs that may function in one of the most critical defense systems for structural and mechanical fitness (Lu et al., 2005).

It is well known that metals such as Cu, Hg, Cd, Fe, and Al have the potential to induce oxidative stress with the generation of •OH in plants (Shahid et al., 2014). miR160 was transcriptionally down-regulated by metals exposure, Cd stress in *B. napus*, Cr stress in rice, Al stress in citrus plants, for example (Huang et al., 2010; Dubey et al., 2020; Zhou et al., 2020). On one hand, the low expression of miR160 causes elevated auxin which was required to combat metals stress, on the other hand, *ARFs* expression enhances adventitious and LR development, which could improve metals tolerance.

In the field, macronutrients and micronutrients are necessary for plants. As a nitrogen-starvation responsive gene, miR160 regulated the establishment of root system architecture through the auxin signaling pathway under nitrogen starvation conditions in plants (Liang et al., 2012; Hua et al., 2020; Singh and Singh, 2021). miR160 could enhance LRs or ARs developments under N-deficiency conditions to maximize the capacity of many plants, such as *Arabidopsis*, cotton, and *B. napus*, to uptake the little available nitrogen (Magwanga et al., 2019). In common beans, miR160 was found to be differentially regulated under P-deficiency in different organs. This was conducive to coping with nutrient deficiency stresses (Valdes-Lopez et al., 2010). Thus, miR160 can be used as an engineering target to improve nutrient usage efficiency and reduce fertilizer application.

### Biotic Stress

Biotic stress leads to severe damage in plants. Bacterial and fungal pathogen exposure could cause induction of miR160 and downregulation of *ARFs* to generate defense responses in *Arabidopsis* (*Pst* DC3000, *Botrytis cinerea*), banana (*Fusarium oxysporum*), and cassava (*Colletotrichum gloeosporioides*) (Zhang et al., 2011; Pinweha et al., 2015; Xue and Yi, 2018; Cheng et al., 2019). miR160 downregulated *ARFs* levels and expressions of auxin-response genes (*AXR3/IAA17*, *BDL/IAA12*, and *GH3-like*), and increased callose deposition to activate basal defense PAMP-triggered immunity (Stork et al., 2010). Furthermore, it was also found that miR160 played a crucial part in local defense and systemic acquired resistance response during potato-*P. infestans* interaction by regulating antagonistic cross-talk between auxin-mediated growth and salicylic acid-mediated defense responses (Natarajan et al., 2018). Moreover, recent studies demonstrated a critical role of miR160 in plant defense against viruses and pests, such as the mosaic virus (Bazzini et al., 2007; Yin et al., 2013; Kundu et al., 2017), rice stripe virus (Du et al., 2011), and brown planthopper (Tan et al., 2020). It may be related to the induction of RNA silencing pathway components.

## Participate in Secondary Metabolism

To our knowledge, plant secondary metabolism is an adaptation of plants to the environment, resulting from the plant interactions between biotic and abiotic factors during the long-term evolution process. There are few studies on the regulation of secondary metabolism by miR160, for example, one study showed that the overexpression of miR160a reduced the *GH3-like* level and negatively regulated the biosynthesis of tanshinones by targeting *ARF10/16/17* in *Salvia miltiorrhiza* hairy roots (Zhang et al., 2020). The miR160-*ARF10/16* pathway also regulated terpenoid indole alkaloid biosynthesis (Shen et al., 2017). Recent research suggested that miR160h-*ARF18* potentially regulated the accumulation of anthocyanins in poplar, but the exact regulation network remained unclear (Wang et al., 2020).

## Conclusion

Plants use a complex variety of transcriptional, post-transcriptional, and translational gene expression programs to survive in the wild. miR160-guided post-transcriptional gene regulation acts a pivotal part in plants. Up to now, rapid and significant progress has been made in miR160 biogenesis, targets prediction, biological functions, and molecular mechanisms. miR160 regulates plant growth and development by interacting with target genes *ARFs*, a regulatory pathway that is closely related to auxin signal transduction (Table 1). miR160, therefore, can act as a new breeding tool in plant genetic improvement to achieve better agronomic characters. Moreover, there may be feedback regulation between miRNAs and their target genes. Studies have shown that a staggering number of miRNA genes were formed under the control of their targets. Their expression level, duplication status, and miRNA–target interaction were important to the evolution of miRNAs and targets. But little research has been done on the co-evolution of miR160 and *ARF* (Carthew and Sontheimer, 2009; Huang et al., 2016; Liu T. et al., 2016).

**TABLE 1:** List of miR160 identified in various plants during their growth and development.

Different plant groups	Species	Target genes	Functions	References
Monocots	Rice (*Oryza sativa*)	*ARF18*	Stem development (plant dwarfing); leaf development (rolled leaves); seed setting, seed width, and weight	Huang et al., 2016
	Maize (*Zea mays*)	*ARF17/19*	Embryogenic calli callus induction; anther development	Li et al., 2019; Lopez-Ruiz et al., 2019
Dicots	*Arabidopsis thaliana*	*ARF10/16/17*	Seed germination (embryo development, SE, hypocotyl elongation); root development (primary root length, lateral root number, root cap formation, AR formation); stem development (plant dwarfing); leaf development (leaf shape defects and leaf symmetry anomalies); callus formation and shoot regeneration; abnormal stamen structure (anther and pollen development) and male sterility; ectopic siliques	Mallory et al., 2005; Wang et al., 2005, 2017; Liu et al., 2007, 2010, 2013; Liang et al., 2012; Qiao et al., 2012; Qiao and Xiang, 2013; Wu et al., 2013; Yang et al., 2013; Liu Z. et al., 2016; Wojcik et al., 2017; Dai et al., 2021
	Longan (*Dimocarpus longan*)	*ARF10/16/17*	SE	Lin et al., 2015
	Soybean (*Glycine max*)	*ARF10/16/17*	Nodule development	Turner et al., 2013; Nizampatnam et al., 2015
	Tomato (*Lycopersicon esculentum*)	*ARF10A/10B/16/17*	Lateral root production; leaf shape and structure, stomatal development, and AQPs expression; pollen development, floral organ patterning, and abscission; early fruit and seed development	Hendelman et al., 2012; Ben-Gera et al., 2016; Damodharan et al., 2016, 2018; Liu X. et al., 2016; Zhao et al., 2019; Keller et al., 2020
	Brassica (*Brassica campestris*)	*ARF17*	Root development; silique length	Chen et al., 2018; Singh and Singh, 2021
	Potato (*Solanum tuberosum*)	*ARF17*	Leaf curvature	Natarajan and Banerjee, 2020
	Poplar (*Populus deltoides*)	*ARF17*	AR development	Liu et al., 2020
	Lotus (*Nelumbo nucifera*)	*ARF17*	AR development	Libao et al., 2020
	Apple (*Malus domestica*)	*ARF16/17*	AR development	Meng et al., 2020
	*Medicago sativa*	*ARF10/16/17*	Root development (primary root length, lateral root number); nodule development	Bustos-Sanmamed et al., 2013
	Cotton (*Gossypium hirsutum*)	*ARF10/17*	Anther development; fiber length	Liu et al., 2019; Chen et al., 2020
	Tobacco (*Nicotiana tabacum*)		Bud and vascular bundle development	Valoczi et al., 2006
	*Phaseolus vulgaris*	*ARF10/16/17*	Seed development	Parreira et al., 2021

Sessile by nature, plants have to suffer different kinds of abiotic and biotic stresses (Figure 1B). Limited by the resources available, plants always need to control the balance between development and stress responses (Fan et al., 2014). It has been demonstrated that transcription factors are important in the trade-off between development and stresses responses (Pajerowska-Mukhtar et al., 2012). miR160 implicates in multiple regulations of biological processes by regulating *ARFs*. For example, as the main root and floral regulator, miR160 and its *ARF* targets were proved to involve in heat stress, drought tolerance and N-deficiency stress (Magwanga et al., 2019; Chen et al., 2020; Dai et al., 2021). By controlling the establishment of the root system architecture of root, the key organ governing water and nutrients uptake, miR160 was able to regulate plants development under N-deficiency condition (Liang et al., 2012). A better understanding of miR160 in stress responses will help us design new strategies to improve the combined stress tolerance of crop plants.

In summary, the miR160-*ARF* module makes up a crucial hub coordinating developments and physiological responses with endogenous and environmental signals. Its universality among plants and diverse possibilities for modification, together make it a highly promising genetic manipulation target for crop breeding. However, overexpressing *ARFs* can cause plant dwarfing and leaf deformities, which affects the utilization of plants whose aerial part is the main source of active ingredients. Hence, there is still a lot of work to be done to exploit its biotechnological potentials, such as a new function mining of miR160, upstream regulatory mechanism, and interaction with other non-coding RNAs (long non-coding RNA, lncRNA, and siRNA). We should pay more attention to the function of miR160 in secondary metabolites, which is the key to the formation of good quality crops, especially medicinal plants.

## Author Contributions

KH and YWa collected the documents and wrote the manuscript. LZ conceived and developed the idea of this review and designed the overall concept. ZZ, YWu, and RC revised the manuscript. All authors contributed to the article and approved the submitted version.

## Conflict of Interest

The authors declare that the research was conducted in the absence of any commercial or financial relationships that could be construed as a potential conflict of interest.

## Publisher’s Note

All claims expressed in this article are solely those of the authors and do not necessarily represent those of their affiliated organizations, or those of the publisher, the editors and the reviewers. Any product that may be evaluated in this article, or claim that may be made by its manufacturer, is not guaranteed or endorsed by the publisher.
